# Study of the expression levels of Hepatocyte nuclear factor 4 alpha and 3 beta in patients with different outcome of HBV infection

**DOI:** 10.1186/1743-422X-9-23

**Published:** 2012-01-18

**Authors:** En-Qiang Chen, Hui Sun, Ping Feng, Dao-Yin Gong, Cong Liu, Lang Bai, Wen-Bing Yang, Xue-Zhong Lei, Li-Yu Chen, Fei-Jun Huang, Hong Tang

**Affiliations:** 1 Center of Infectious Diseases, West China Hospital of Sichuan University, Chengdu 610041, Sichuan Province, People's Republic of China; 2 Division of Infectious Diseases, State Key Laboratory of Biotherapy, Sichuan University, Chengdu 610041, People's Republic of China; 3 Department of Infectious Diseases, North Sichuan Medical College, Nanchong 637000, People's Republic of China; 4 Department of Forensic Pathology, College of Basic medicine and Forensic medicine, Sichuan University, Chengdu 610041, Sichuan Province, People's Republic of China

**Keywords:** Hepatocyte nuclear factor-4 alpha, Hepatocyte nuclear factor-3 beta, Chronic hepatitis B, Severe hepatitis B, Liver cirrhosis, Immunohistochemistry

## Abstract

Hepatocyte nuclear factors 4 alpha (HNF4α) and 3 beta (HNF3β) are members of a group of liver-enriched transcription factors (LETFs) that play important roles in regulating the replication of hepatitis B virus (HBV) and liver inflammation. However, the relationship of the level of HNF4α and HNF3β with the severity of HBV-infected liver diseases is unclear. In this study, liver tissue samples from different types of HBV patients were collected, and HNF4α and HNF3β expression were detected by immunohistochemistry. The expression of HNF4α was significant higher in patients with severe hepatitis B(SHB) than those with chronic hepatitis B(CHB) and liver cirrhosis(LC) (both *P *< 0.05), but similar between patients with CHB and LC (*P *> 0.05). And the expression of HNF3β was similar among patients with CHB, LC and SHB (*P *> 0.05 for all pairwise comparison). This suggests that the expression level of HNF4α was different in patients with different outcome of HBV infection, high expression level of HNF4α may correlate with occurrence of SHB

## Background

Hepatitis B virus (HBV) infection is a serious global health problem, with 2 billion people infected worldwide [1], and it would lead to various clinical outcomes, ranging from an asymptomatic carrier state (inactive HBV carrier) to acute or chronic liver disease including chronic hepatitis B (CHB), severe hepatitis B (SHB), liver cirrhosis (LC) and hepatocellular carcinoma(HCC) [2]. Because of the lack of specific therapies, those patients are at high risk of mortality [3]. Currently, HBV infection has become the 10th cause of death worldwide, and approximately 15-40% of those patients with CHB will develop to end-stage liver disease including LC, SHB, or HCC [1].

Outcomes of HBV infection are affected by both virus (such as: virus variation, virus protein, virus genotype, etc.) and host factors (such as: cellular immunity, cytokines, apoptosis, etc.) [4-8], and evidence have showed that viral replication is mainly correlated to the development or progression of diseases [2,4]. In fact, viral replication is not only regulated by host factors [9], but also can cause changes in the level and activity of host factors, which would result to severe hepatic damage due to cell dysfunction. Hepatic nuclear factors (HNFs) are a group of important host transcription factors that mainly reside in the liver and regulate numerous liver-expressed genes [10]. One of our previous work suggested that HNF4α and HNF3β likely participated in HBV replication in patients with HBV infection, or that HBV replication may somehow influence the expression of hepatocyte nuclear factor 4alpha (HNF4α) and 3 beta (HNF3β) in the liver [11]. Using cell culture and animal models, we also found that HNF4α supports HBV replication in non-hepatic cells and HNF3β inhibits HBV replication [12]. And in recent years, the study of HNF in hepatitis B has become a research hotspot.

Recently some scholars using gene chip analyzed liver tissue samples of HBV infected patients, and they found that HBV infection could increase the transcription of HNF, which may be related to the occurrence of liver injury [13]. So we hypothesized the interaction of HNFs and HBV transcriptional regulation may play an important role in the pathogenesis of disease progression. However, it is still unclear whether the expression level of HNFs is correlated to the clinical outcomes of HBV infection. In present study, we studied the variation of HNF4α and HNF3β expression in different types of hepatitis B, so as to investigate the correlation of HNFs expression and disease severity.

## Results

Liver specimens from 19 HBV infections with variety of clinical outcomes were studied, comprised of 6 patients with chronic hepatitis B (Group A), 6 patients with liver cirrhosis (Group B) and 7 patients with severe hepatitis B (Group C). The serum HBV DNA levels of those patients were ranged from 3 to 5 log10 copies/mL, and its level was comparable among group A, B and C. Detailed characteristics of those patients were summarized in Table 1.

**Table 1:** Demographic and clinical characteristics

	CHB (Group A)	LC (Group B)	SHB (Group C)
**Number**	6	6	7

**Age, ys**	42.67	45.50	36.86

**Male/Female**	6/0	4/2	4/3

**HBeAg(+)/HBeAg(-)**	0/6	1/5	0/7

**HBV DNA, log10 copies/mL**	3.60	3.55	3.29

### Expression levels of HNF4α

**Figure 1:**
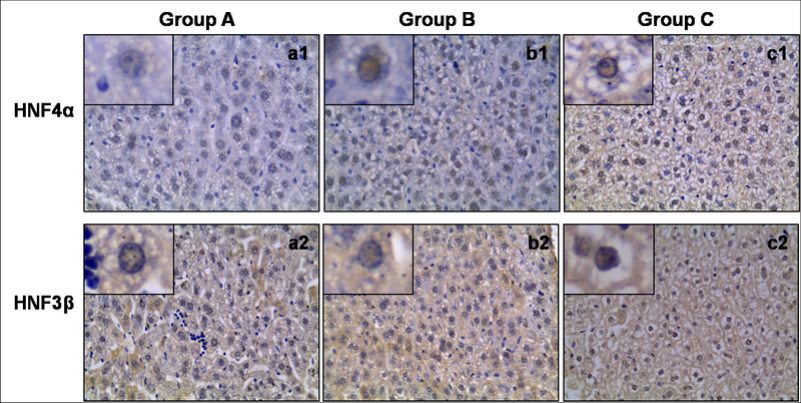
**The expression of HNF4α and HNF3βin liver tissue of typical patients in three groups were shown**. The scores of HNF4α were higher in c1 as compared to a1 and b1; while the scores of HNF3 were similar among a2, b2 and c2.

Expression of HNF4α was detected in all 19 specimens, and the typical immunohistochemical images of HNF4α in these groups were shown in Figure 1. The mean scores of HNF4α were 4.29, 4.75 and 5.58 for specimens in group A, B, and C, respectively (Table 2). Among those groups, HNF4α scores was significant higher in group C as compared with either group A or B (Both *P *< 0.05). Though higher scores were also observed in group B, the difference was not statistic significantly as compared with group A.

**Table 2:** Scores of the expression levels of HNF4α

	CHB (Group A)	LC (Group B)	SHB(Group C)
**Pat.1**	2.5	4.5	6.0

**Pat.2**	4.5	5.0	6.0

**Pat.3**	5.0	5.0	6.0

**Pat.4**	4.5	5.0	6.0

**Pat.5**	4.0	4.5	4.5

**Pat.6**	5.0	4.5	5.0

**Pat.7**	4.5	-	-

**Mean**	4.29^▲^	4.75^Δ^	5.58^▲Δ^

The symbol "▲" and "Δ" indicated significant difference between two groups (*p *< 0.05)

These data showed that HNF4α expression was increased significantly in patients with SHB, and suggested that high expression of HNF4α may correlate with occurrence of SHB

### Expression levels of HNF3β

Expression of HNF3β was detected in all 19 samples, and the typical immunohistochemical images of HNF3β in these groups were shown in Figure 1. The mean scores of HNF3β were 2.71, 2.33 and 2.50 for specimens in group A, B, and C, respectively (Table 3). Though more scores were observed in group A, but the difference was not significant as compared with either group B or C. And the difference of score between group B and C was also not significant.

**Table 3:** Scores of the expression levels of HNF3β

	CHB (Group A)	LC (Group B)	SHB (Group C)
**Pat.1**	2.5	1.5	3.0

**Pat.2**	2.0	3.0	3.0

**Pat.3**	2.0	1.0	1.5

**Pat.4**	4.5	0.5	1.5

**Pat.5**	3.0	5.5	1.0

**Pat.6**	3.0	2.5	1.0

**Pat.7**	2.0	-	-

**Mean**	2.71	2.33	2.50

These data showed HNF3β expression was similar between different groups, and suggested that the expression of HNF3β may not correlate with clinical outcomes of HBV infections.

## Discussion

Current study reported that abnormal expression of host-related genes in hepatitis B patients played important roles in the occurrence, development and prognosis of liver damage [6], and those host-related genes mainly were apoptosis and inflammation-associated genes [14-16]. Recently some scholars using gene chip analyzed liver tissue samples of HBV infected patients, and they found that HBV infection increased the transcription of many genes such as HNF4, retinoid X receptor and peroxisome proliferator-activated receptors (RXR/PPARs) and CCAAT/enhancer binding protein (C/EBP), and so on [13may correlate with disease severity of HBV infections. In contrast, HNF3β expression was similar among patients with different outcome of HBV infection, and it suggested HNF3β expression was not associated with disease severity caused by HBV, though our previous work showed HNF3β could inhibit HBV replication [was higher in liver tissue of patients with SHB, while the expression of HNF3β was similar among liver tissue of patients with different clinical outcomes, and our findings indicated that the expression level of HNF4α and HNF3β in different HBV infections, and we found that the expression of HNF4α ], which suggested the high expression of those gene may be related to the occurrence of liver injury caused by HBV. In present study, we observed the expression of HNF4α [17,18].

HNFs are a class of protein molecules that mainly exist in liver and regulate liver-specific gene expression at transcriptional level, and HNF4α is one of the most important regulatory factors of HNFs [10]. It has been reported that the over-expression of fibrinogen-like protein 2(fgl2) is correlated with occurrence of SHB, and the gene promoter of fgl2 would be combined and activated by HNF4α, and it suggested that HNF4α may play an important role in the occurrence of SHB [19]. Recently, we inhibited the expression of HNF4α in mice with acute liver failure induced by lipopolysaccharide and D-Galactosamine, and we found that the liver damage of mice with LF was alleviated and mortality rate was significant decreased (unpublished data). The findings from experimental data also indicated that the over-expression of HNF4α was an important factor for the occurrence of liver failure, which was consistent with the findings observed in liver tissue samples of patients with SHB in present study.

It has reported that HNF could bind and activate the four promoters of HBV genome, which is important for the regulation of transcription and replication of HBV [20]. So it is reasonable to infer that the correlation of over-expression of HNF4α with severe hepatitis B may associate with its effect on HBV. We previously analyzed the incidence of mutations in HBV precore/basal core promoter (BCP) in patients with SHB, and found combined mutation of A1762T + G1764A (TA mutation) in HBV precore/BCP region might be related to the aggravation of chronic HBV infection(unpublished data), because of enhanced transcription and replication capacity of HBV [20]. Interesting, evidence showed that the binding site of HNF4α on HBV also located in HBV precore/BCP region, so TA mutation in this region may affect HNF4α on regulation of HBV transcription and replication [20]. In fact, the results from non-liver derived cell lines and HBV replication mouse model (with/without HNF4α inhibition) had confirmed that HNF4α supported high replication level of TA mutation, [20] which may contribute to the aggravation of hepatitis B. Considering the positive correlation between HNF4α expression and HBV replication in liver tissue of IHBV infections, and avoiding the interference of HBV DNA level on HNF4α expression, only patients with serum HBV DNA less than 5 log10 copies/mL were included, and the mean level of HBV DNA was comparable between each group in this study. Thus, we believe our result is a credible finding.

In summary, our results suggested that the expression level of HNF4α was different in patients with different outcome of HBV infection, and high expression level of HNF4α may correlate with occurrence of SHB.

## Materials and methods

### Study population

A total of 19 liver tissue samples from 19 patients were obtained from Hepatobiliary Surgery Department and Center of Infectious Diseases, West China Hospital of Sichuan University, between 2007 and 2008. The clinical outcomes of those samples were consisted of CHB (group A, n = 6), LC (group B, n = 6) and SHB (group C, n = 7).

All patients were positive for hepatitis B surface antigen (HBsAg) for at least 6 months, were without co-infection of human immune-deficiency virus (HIV) and other hepatitis viruses(hepatitis A, C and E virus), and were without auto-immune hepatitis, drug-induced hepatitis, and liver damages caused by other etiologies.

The study protocol was conformed to the ethical guidelines of the 1975 Declaration of Helsinki.

### Preparation of integrated tissue slice slides

Two different types of liver tissue specimens were obtained: wedge and needle biopsy specimens. The wedge specimens (2 × 2 × 2 cm^3^) were taken during open surgery, and needle biopsy specimens were obtained by a biopsy needle under ultrasound guidance. Liver specimens were fixed in 10% formalin for 24 to 48 h and embedded in paraffin.

For wedge specimens, a blank paraffin block was prepared and used as the recipient for the tissue samples, and a 2 mm punch needle was used to punch the donor paraffin block to transfer donor cores into blank recipient blocks at a defined region according to the marker made on blank paraffin block in advance. After all individual specimens were integrated on a single paraffin block, the integrated tissue was sectioned at a slice thickness o f 4 mm. Sections were stretched in a 40°C water bath, mounted a glass slides, and dried at 60°C for 2 h.

For needle biopsy specimens, paraffin-embedded tissue was sectioned at a slice thickness of 4 um. And all sections were stretched in a 40°C water bath and mounted a glass slides at a defined region according to the marker made on the slide in advance. As a result, all tissue slices were integrated on a single slide.

### Detection of HNF4α and HNF3β in liver tissue specimen by immunohistochemistry

In this study, immunohistochemistry was used to detect expression levels of HNF4α and HNF3β in liver tissue specimens. Paraffin sections were deparaffinized, rehydrated and incubated with 30 ml/L H_2_O_2 _at 37°C for 15 min. Antigen retrieval was achieved using Supersonic wave repair for 30 sec (for detection of HNF4α) or microwave repair for 3 times plus Supersonic wave repair for 30 sec (for detection of HNF3β). Incubation with primary antibodies was performed at 37°C for 1 h and 4°C overnight, followed by three washes with phosphate-buffered saline (PBS). The primary antibodies used to detect the expression of HNF4α and HNF3β were goat anti-human HNF4α polyclonal antibody (1:50 diluted) (Santa Cruz, USA), and goat anti-human HNF3β polyclonal antibody (1:30 diluted) (Santa Cruz, USA), respectively. All sections were then incubated with biotin-labeled secondary antibodies including rabbit anti-goat IgG. After washing 3 times with PBS, the immune complexes in the specimens were detected using DAB Substrate Kit (Wuhanboster Biological Technology, China) according to the manufacture's instructions. The slides were then counterstained with hematoxylin before being mounted.

### Immunohistochemical scoring criteria

The immunohistochemical results of integrated tissue slice slides were read independently by two pathologists who had no prior knowledge of the experimental procedures. The percentage of positive cells and the positive staining intensity were checked and scored using the Axiotis score standard by observation of 5 randomly chosen fields at 400-fold magnification. Sections detected by immunohistochemistry were stained with DAB and conterstained with hematoxylin, and positive signals were manifested as yellow, brown, or tan staining. The scoring criteria for the proportion of positive cells were: 0 = 0%-10% positive cells; 1 = 11%-25% positive cells; 2 = 26%-50% positive cells; 3 = 51%-75% positive cells; 4 = 76%-100% positive cells. The scoring criteria for staining intensity in positive cells were: 0 = no color, 1 = yellow, 2 = brown, 3 = tan. The sum of the scores for proportion of positive cells and staining intensity was the final score for the sample. If the film-reading results of the two pathologists were different for the same sample, we then used the mean of the scores to generate the final result.

### Statistical analysis

The scores of HNF4α and HNF3β in specimens in each group were presented as mean, and data were analyzed using the SPSS version 13.0 software package (SPSS, Inc., Chicago, IL). Tests of significance were 2-tailed, with significance being indicated by a *P*-value < 0.05.

## Competing interests

The authors declare that they have no competing interests.

## Authors' contributions

We thank Prof. X-HY from Hepatobiliary Surgery Department for providing wedge specimens to this study. All authors read and approved the final manuscript
